# Missing mass approximations for the partition function of stimulus driven Ising models

**DOI:** 10.3389/fncom.2013.00096

**Published:** 2013-07-24

**Authors:** Robert Haslinger, Demba Ba, Ralf Galuske, Ziv Williams, Gordon Pipa

**Affiliations:** ^1^Martinos Center for Biomedical Imaging, Massachusetts General HospitalCharlestown, MA, USA; ^2^Department of Brain and Cognitive Sciences, Massachusetts Institute of TechnologyCambridge, MA, USA; ^3^Systems Neurophysiology, Department of Biology, Technische Universität DarmstadtDarmstadt, Germany; ^4^Department of Neurosurgery, Massachusetts General HospitalBoston, MA, USA; ^5^Department of Neuroinformatics, Institute of Cognitive Science, University of OsnabrueckOsnabrueck, Germany

**Keywords:** Ising model, stimulus coding, population codes, partition function, multiple unit recordings, network function

## Abstract

Ising models are routinely used to quantify the second order, functional structure of neural populations. With some recent exceptions, they generally do not include the influence of time varying stimulus drive. Yet if the dynamics of network *function* are to be understood, time varying stimuli must be taken into account. Inclusion of stimulus drive carries a heavy computational burden because the partition function becomes stimulus dependent and must be separately calculated for all unique stimuli observed. This potentially increases computation time by the length of the data set. Here we present an extremely fast, yet simply implemented, method for approximating the stimulus dependent partition function in minutes or seconds. Noting that the most probable spike patterns (which are few) occur in the training data, we sum partition function terms corresponding to those patterns explicitly. We then approximate the sum over the remaining patterns (which are improbable, but many) by casting it in terms of the stimulus modulated missing mass (total stimulus dependent probability of all patterns not observed in the training data). We use a product of conditioned logistic regression models to approximate the stimulus modulated missing mass. This method has complexity of roughly *O*(LNN_pat_) where is *L* the data length, *N* the number of neurons and *N*_pat_ the number of unique patterns in the data, contrasting with the *O*(*L2*^*N*^) complexity of alternate methods. Using multiple unit recordings from rat hippocampus, macaque DLPFC and cat Area 18 we demonstrate our method requires orders of magnitude less computation time than Monte Carlo methods and can approximate the stimulus driven partition function more accurately than either Monte Carlo methods or deterministic approximations. This advance allows stimuli to be easily included in Ising models making them suitable for studying population based stimulus encoding.

## 1. Introduction

The role, if any, that spike timing correlations between neurons play for neural encoding of stimuli remains unclear (Abbott et al., 1999; Nirenberg et al., 2001; Averbeck et al., 2003, 2006; Chelaru and Dragoi, 2008; Jacobs et al., 2009; Josic et al., 2009). This is often studied by fitting statistical models to population data and comparing the encoding properties of models which include correlations, and thus the collective code, with models that only include the independent stimulus drive to each neuron. Fitting such correlated models is not trivial. One extremely successful model which includes both time varying stimuli and lagged spike timing correlations between neurons is the cross-coupled Generalized Linear Model (GLM) (Okatan et al., 2005; Pillow et al., 2008; Truccolo et al., 2010; Gerhard et al., 2011). This approach fits each neuron's spikes independently as a function of stimuli, but also conditioned upon the *past spiking history* of all other neurons in the population. The conditional independence assumption follows from causality, a neuron at time *t* can only be influenced by events in the past, at time *t*′ < *t*. Conditional independence makes fitting coupled GLMs computationally tractable, since each of the *N* neurons's spikes can be fit separately using efficient iteratively reweighed least squares algorithms (McCullagh and Nelder, 1989; Komarek and Moore, 2003; Komarek, 2004). As they include both stimuli and time lagged interactions between neurons, GLMs often provide an extremely good description of how populations collectively code dynamic stimuli.

GLMs do not, however, include dependencies between neurons *in the same time bin*. If time bins are small, on the order of a millisecond, the conditional independence assumption will hold. However, for some applications, larger time bins may be of interest. For example the stimulus might have a slower time scale, or it might not matter if spikes from two neurons arrive at a downstream neuron with millisecond precision. Thus one might be interested in the probabilities of certain patterns or “code words” across the population with those patterns defined at longer (10 s of ms) time scales. For these larger bin sizes correlations within the same time bin may matter. A standard approach for fitting second order correlated models where the correlations are between neurons in the same time bin is the Ising model (Martignon et al., 2000; Schneidman et al., 2006; Tang et al., 2008; Roudi et al., 2009b; Ganmor et al., 2011). However, the Ising model is computationally intensive to evaluate. Ensuring that the probabilities of all possible code words sum to 1 requires the explicit calculation of a normalization factor or *partition function* obtained by summing terms over all possible code words which scale as 2^*N*^. Such normalization is crucial for performing model comparisons. For example deducing the importance of correlations between neurons by comparing the Ising model goodness of fit to a model that does not include couplings between neurons. It is also important for accurately calculating information theoretic quantities such as entropy.

Various approximate techniques for calculating the partition function, either involving some type of Monte Carlo importance sampling (Broderick et al., 2007; Salakhutdinov, 2008) or deterministic variational approximations such as mean field theories or the Bethe approximation have been developed. However, Monte Carlo techniques generally require a significant amount of computation time and variational methods can be inaccurate, exhibiting significant bias since they provide lower bounds on the partition function. In part for such reasons, until recently (Tkacik et al., 2010; Granot-Atedgi et al., 2013), Ising models only modeled the *stationary* distribution of firing rates and correlations, despite the fact that stimulus drive is often a much stronger influence on a neuron's spike probability than the correlations between neurons. Although stimulus drive is formally simple to include in Ising models, such stimulus driven models take an extremely long time to evaluate, because the partition function must be separately recalculated for every unique value of the stimulus observed in the data. Partition function computation time now potentially scales as 2^*N*^ × *L* where *L* is the data length (number of time bins).

Here we present a method for quickly (in minutes or less) and accurately (with low bias and variance) calculating the partition function of stimulus driven Ising models *over the entire length of a data set*. This method is based upon a simple observation: for population spiking data most of the possible patterns are extremely improbable. Thus their corresponding terms in the partition function contribute little, rather it is the high probability patterns, most of which appear in the training data, that dominate the partition function. In general these patterns will be few, numbering *N*_pat_ << 2^*N*^. Therefore we propose to explicitly sum only these *N*_pat_ terms and approximate the remainder of the sum by estimating the stimulus varying *missing probability mass*. The missing mass is the total probability of all patterns that do not appear in the data and will be small for real neural populations which spike sparsely. Thus an approximation will be sufficient to correct the partition function.

We show that the stationary (not stimulus variable) missing mass can be approximated using simple counting via the Good Turing estimate (Good, 1953; Orlitsky et al., 2003) and that the stimulus driven missing mass can be well approximated using a product of conditioned (upon other neurons spikes in the same time bin) logistic regression models. The computation time of this procedure scales approximately as *O*(LNN_pat_). For most data sets this translates to minutes or seconds, as opposed to Monte Carlo importance sampling (which can take hours) or naive summation (which is intractable for large populations). Moreover, as we demonstrate using both simulated data and *in vivo* recorded population data, our method provides extremely accurate estimates of the stimulus modulated partition function, in contrast to deterministic methods (which can have large bias), often leading to pattern probability distributions normalized within less than a tenth of a percent.

## 2. Materials and methods

Ising models describe the probability of any pattern of spikes $\vec{\sigma }$ across neurons as

(1) $$P(\vec{\sigma })=\frac{e^{\vec{h}\cdot \vec{\sigma }\;+\;{\vec{\sigma }}^{⊤}J\vec{\sigma }}}{Z}$$

where $\vec{h}$ is a fitted parameter vector describing the stimulus drive to each neuron, and *J* is a fitted parameter matrix describing the coupling between neurons (see Appendix A). Since the numerator is not guaranteed to give a normalized probability distribution over all possible patterns, an explicit normalization or *partition function Z* is introduced.

(2) $$Z=\sum_{\vec{\sigma }}e^{\vec{h}\cdot \vec{\sigma }\;+\;{\vec{\sigma }}^{⊤}J\vec{\sigma }}$$

*Z* is extremely time consuming to evaluate because involves a sum over all possible patterns which scale as 2^*N*^. The situation is even worse if the Ising model is *stimulus dependent*:

(3) $$P(\vec{\sigma }|s)=\frac{e^{\vec{h}(s)\cdot \vec{\sigma }\;+\;{\vec{\sigma }}^{⊤}J\vec{\sigma }}}{Z(s)}$$

where $\vec{h}(s)=\{h_{1}(s),h_{2}(s)\ldots h_{N}(s)\}$ is now a vector of functions of the stimulus. Explicit functional forms for this vector will be experiment dependent and we will present several in section 3. Here we merely note that each element of this vector can often be written as a linear sum of stimulus dependent basis functions multiplied by fitted parameters, or equivalently, the multiple of a stimulus covariate matrix *C*(*s*) multiplied by a fitted parameter matrix β, such that $\vec{h}(s)=C(s)\beta$. The crucial point is that if stimulus drive is included, the partition function *Z*(*s*) has to be evaluated for all unique observed values of the stimulus *s*. Even if the parameters $\vec{h}(s)$ and *J* are known, a naive evaluation of *Z*(*s*) can take hours or days for an entire data set.

In this paper we present a fast and accurate approximation for *Z*(*s*). Assume for the moment that $\vec{h}(s)$ and *J* are known and we wish to calculate *Z*(*s*) given these parameters. The crucial insight is that while *Z*(*s*) involves a sum over all possible patterns, the patterns that appear in the training data are most probable. Another way to say this is that patterns with many spikes are highly improbable, because population spiking tends to be sparse. Thus *Z*(*s*) can be split into two terms.

(4) $$Z(s)=X(s)+Y(s)=\sum_{{\vec{\sigma }}_{T}}e^{\vec{h}(s)\cdot \vec{\sigma }\;+\;{\vec{\sigma }}^{⊤}J\vec{\sigma }}+\sum_{{\vec{\sigma }}_{\notin T}}e^{\vec{h}(s)\cdot \vec{\sigma }\;+\;{\vec{\sigma }}^{⊤}J\vec{\sigma }}$$

where ${\vec{\sigma }}_{T}$ denotes the set of patterns observed in the training data and ${\vec{\sigma }}_{\notin T}$ denotes the patterns that are not observed in the training data. Since in general $|{\vec{\sigma }}_{T}|<<2^{N},$, *X*(*s*) will be quick to evaluate exactly. The goal is to approximate *Y*(*s*).

Approximating *Y*(*s*) requires estimating the stimulus dependent *missing mass*, e.g., the total stimulus dependent probability mass of patterns not observed in the training data.

(5) $$M(s)=\sum_{\sigma_{\notin T}}P(\sigma |s)$$

For the Ising model this is

(6) $$M(s)=\frac{\sum_{{\vec{\sigma }}_{\notin T}}e^{\vec{h}(s)\cdot \vec{\sigma }\;+\;{\vec{\sigma }}^{⊤}J\vec{\sigma }}}{Z}=\frac{Y(s)}{X(s)+Y(s)}$$

and thus *Y*(*s*) may be obtained by simple inversion.

(7) $$Y(s)=\frac{M(s)}{1-M(s)}X(s)$$

### 2.1. Good turing estimate for the static missing mass

Before considering how the missing mass is modulated by stimuli, we discuss how to estimate its average across the stimulus distribution: $\bar{M}=\int P(s)M(s)ds$. For moderately sized neuronal populations $\bar{M}$ can be evaluated by fitting a *stimulus independent* Ising model and explicitly summing terms analogously to Equation 4. For larger populations, however, this becomes less tractable. Fortunately, an unbiased estimate of the stationary (stimulus independent) missing mass can be obtained using the *Good Turing* estimate (Good, 1953; Orlitsky et al., 2003). Originally developed by Alan Turing during his efforts to crack the Enigma code (Good, 2000), this estimates the total summed probability of all patterns not observed in the training data by counting the unique patterns observed *only once* in the training data and dividing this value by the total number of observations in the training data

(8) $$M_{\text{GT}}=\frac{\left|{\vec{\sigma }}_{\text{occur once}}\right|}{L}$$

This estimator is common in many fields, for example in biological censuses, where it is used to estimate the probability of all animal species not observed (Good, 1953; Dornelas et al., 2013). It is unbiased (Good, 1953) and it can be shown that tight bounds on the estimated missing mass exist (McAllester and Schapire, 2000; Berend and Kontorovich, 2012). As we will show empirically below, the Good Turing estimate is extremely accurate for neuronal population data despite merely requiring simple counting of training data patterns.

If one uses the Good Turing approximation, then one is assuming that the missing mass is not modulated by the stimulus, i.e., $M(s)=\bar{M}=M_{\text{GT}}$. This corresponds to assuming that *X*(*s*) and *Y*(*s*) covary with the stimulus in the same way. That is: $X(s)=\bar{X}\xi (s)$ and $Y(s)=\bar{Y}\xi (s)$ and thus

(9) $$M(s)=\frac{\bar{Y}\xi (s)}{\bar{X}\xi (s)+\bar{Y}\xi (s)}=\frac{\bar{Y}}{\bar{X}+\bar{Y}}=\bar{M}$$

We call calculation of *Z*(*s*) using the Good-Turing missing mass the *Good-Turing Approximation*. We emphasize that in cases of strongly varying stimulus drive, this will likely *not* be a good approximation. Specifically, as we will show in the results, using a constant missing mass corrects for the bias in our estimate of *Z*(*s*), but not the variance. This is because *X*(*s*) and *Y*(*s*) are comprised of different patterns and thus will not necessarily vary with the stimulus in the same way. That is $X(s)=\bar{X}\xi_{X}(s)$ and $Y(s)=\bar{Y}\xi_{Y}(s)$ where ξ_*X*_(*s*) ≠ ξ_*Y*_(*s*) necessarily. We remedy this in the next section, where we show how to calculate the *stimulus modulated* missing mass.

### 2.2. Stimulus modulated missing mass

The central insights for approximating *M*(*s*) are threefold. First, if the joint probabilities *P*(σ_1_, σ_2_ … σ_*N*_|*s*) are known, then the missing mass can be found by simply summing over patterns observed in the training data, which are relatively few.

(10) $$M(s)=1-\sum_{{\vec{\sigma }}_{T}}P(\sigma_{1},\sigma_{2}\ldots \sigma_{N}|s)$$

Second, these joint probabilities can be written as products of conditional probabilities, e.g.,

(11) $$\begin{array}{l} P(\sigma_{1},\sigma_{2}\ldots \sigma_{N};s)=P(\sigma_{1}|\sigma_{2}\ldots \sigma_{N};s)\\ \;P(\sigma_{2}|\sigma_{3}\ldots \sigma_{N};s)\ldots P(\sigma_{N}|s) \end{array}$$

The third insight is that these conditional probabilities can be approximated using logistic regression models, at least to the extent required to obtain a good estimate of the stimulus modulated missing mass and partition function.

Logistic regression has long been used to approximately fit Ising models because the Ising conditional probabilities are exactly given by logistic functions, e.g.,

(12) $$P(\sigma_{i}|{\vec{\sigma }}_{j\;\neq \;i};s)=\frac{\exp[h_{i}(s)+2\sum_{j\;\neq \;i}\sigma_{j}J_{ji}]\sigma_{i}}{1+\exp[h_{i}(s)+2\sum_{j\;\neq \;i}\sigma_{j}J_{ji}]}$$

This result is easily found by expanding the sums in the exponent of the Ising model numerator and absorbing all terms which do not depend upon σ_*i*_ into the partition function (Pawitan, 2001). Independently fitting multiple conditional logistic regression models (one for each σ_*i*_) and equating the fitted parameters with those of the Ising model's joint distribution is the *pseudo likelihood* approach for Ising model fitting (Besag, 1974, 1975) and see Appendix A. While this often gives good parameter estimates, it does not provide a normalized probability distribution. e.g., the product of the conditionals is not in general normalized.

(13) $$\sum_{\vec{\sigma }}\left[\prod_{i\;=\;1}^{N}P(\sigma_{i}|{\vec{\sigma }}_{j\;\neq \;i};s)\right]\neq 1$$

and full distribution normalization again requires evaluating the partition function.

However, the product of conditional probabilities in Equation 11 *is* normalized. Unfortunately, these conditional probabilities are not given by logistic regression models with the same parameters as the Ising model. That is

(14) $$P(\sigma_{i}|\sigma_{j};s)\neq \frac{\exp[h_{i}(s)+2\sigma_{j}J_{ji}]\sigma_{i}}{1+\exp[h_{i}(s)+2\sigma_{j}J_{ji}]}$$

for an arbitrary coupling matrix *J*. Formally, this property, that the marginals of the conditional probabilities do not have the exact same logistic form with the exact same parameters is called lack of *projectivity* (Shalizi and Rinaldo, 2012). In fact, it can be shown that distributions in the exponential family are, in general, not projective.

Fortunately, the exact parameters are not required to estimate the missing mass, merely reasonably accurate estimates of the conditional probabilities of Equation 11. Therefore we will in fact estimate these conditional probabilities using logistic regression, *but not require the parameters to match those of the full Ising model joint distribution*. That is, we will fit conditional logistic regression models

(15) $$P_{\text{CL}}(\sigma_{i}|{\vec{\sigma }}_{j\;>\;i};s)=\frac{\exp[l_{i}(s)+2\sum_{j\;\neq \;i}\sigma_{j}K_{ji}]\sigma_{i}}{1+\exp[l_{i}(s)+2\sum_{j\;\neq \;i}\sigma_{j}K_{ji}]}$$

but *l*_*i*_(*s*) ≠ *h*_*i*_(*s*) and *K*_*ji*_ ≠ *J*_*ji*_ necessarily and the subscript “CL” denotes that these probabilities are given by conditioned (on subsets of neurons) logistic regression models. There are *N*! possible orderings of neurons which could be used in Equation 11. Since neurons with low firing rates will not have sufficient information in their spike trains to deduce the influence of other neurons upon them, we order the neurons in Equation 11 by mean firing rate. That is, we use the lowest firing rate neuron for *P*(σ_*N*_|*s*) and the highest for *P*(σ_1_|σ_2_ … σ_*N*_;*s*).

It is important to note that for the purpose of calculating the missing mass, the fact that *l*_*i*_(*s*) ≠ *h*_*i*_(*s*) and *K*_*ji*_ ≠ *J*_*ji*_ is irrelevant. It is only important that decent estimates of the conditional probabilities be obtained so that they can be used to approximate the stimulus modulated missing mass using Equations 10 and 11. We denote this missing mass estimate as *M*_CL_(*s*). Our procedure for approximating the stimulus driven partition function *Z*(*s*) can be stated as follows:

1. Identify the set of all unique patterns in observed the training data and call this set σ_*T*_.
2. Explicitly calculate $X(s)=\sum_{{\vec{\sigma }}_{T}}e^{\vec{h}(s)\cdot \vec{\sigma }\;+{\vec{\sigma }}^{⊤}J\vec{\sigma }}$.
3. For a population of *N* neurons, independently fit *N* logistic regression models for the conditional probabilities $P_{\text{CL}}(\sigma_{i}|{\vec{\sigma }}_{j>i};s)$. (Exact expression given in Equation 15).
4. Use the conditional probabilities *P*_CL_ to approximate $P(\vec{\sigma }|s)$ for each unique pattern in the training data according to Equation 11 and then approximate the stimulus modulated missing mass *M*_CL_(*s*) using Equation 10.
5. Calculate *Y*(*s*) = *X*(*s*)[*M*_CL_(*s*)/(1 − *M*_CL_(*s*))] and *Z*_CL_(*s*) = *X*(*s*) + *Y*(*s*).

This procedure, which we will refer to as the *conditional logistic* approximation for *Z*(*s*) can accurately estimate the stimulus driven partition function with error of a few tenths of a percent if the mean missing mass $\bar{M}$ is small (a few percent or less). This corresponds to populations which spike sparsely, and for which the number of unique patterns is relatively few (thousands). Throughout this paper, we quantify error using the distribution (over stimuli) of the *ratio* of the estimated *Z*(*s*) over the true partition function *Z*_exact_(*s*) (obtained through exact summation). This ratio is the same for all patterns, i.e., *P*_exact_(σ|*s*)/*P*_CL_(σ|*s*) = *Z*_CL_(*s*)/*Z*_exact_(*s*) and gives the stimulus dependent fraction by which all pattern probabilities are under or over-estimated. Generally we present the 99% bounds (0.005 and 0.995 quantiles) of the distribution.

**Figure 1:**
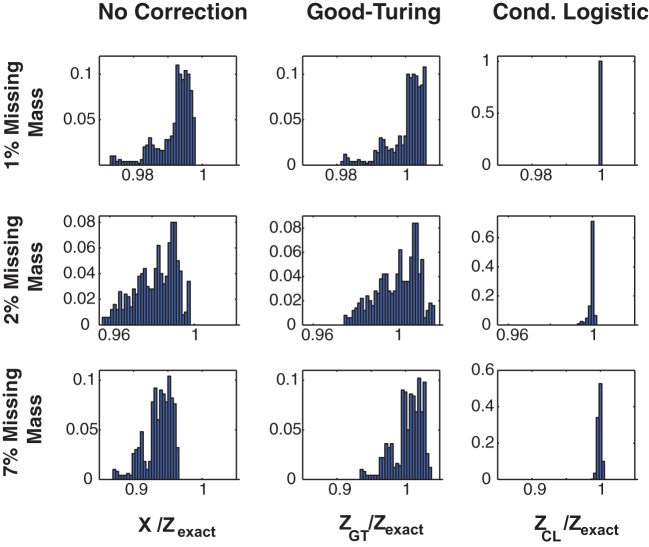
**Three approximations for *Z*(*s*)**. Distributions of *Z*(*s*)/*Z*_exact_(*s*) for three 20 neuron simulations with different missing masses (rows). **Left column:** No correction, e.g., *X*(*s*). **Middle column:** Good-Turing approximation. **Right column:** conditional logistic approximation. While the Good-Turing approximation corrects the distribution's bias, the conditional logistic approximation is required to eliminate the variance.

Figure 1 illustrates the effectiveness of the method. Here we show the error distribution over a time varying stimulus for 3 Ising model simulated, 100 s long data sets (in different rows) constructed to have missing masses of (1, 2, and 7%). (Details of the simulations, and further simulated results are given in section 3 and Figures 3, 4.) We show error distributions for (1) *X*(*s*) only (no correction), (2) the Good-Turing approximation, and (3) the conditional logistic approximation (in different columns). Prior to making any corrections [i.e., approximating *Z*(*s*) by *X*(*s*)] the error distribution has both high bias and high variance. 99% bounds are {0.9738, 0.9973}, {0.9577, 0.9980}, and {0.8723, 0.9649} for the 1, 2, and 7% data, respectively. The Good Turing correction removes the bias, (means of 1.002, 1.000, and 1.003, respectively) but the variance (due to the time varying stimulus) is still large (99% bounds are {0.9820, 1.0059}, {0.9754, 1.0165}, and {0.9352, 1.0345}, respectively). However, the full conditional logistic correction accounting for stimulus modulation removes both the bias and the variance (99% bounds {0.9999, 1.0001}, {0.9938, 1.0009}, and {0.9927, 1.0034}, respectively). The conditional logistic approximation is thus accurate to within a few tenths of percent even if the missing mass is relatively large (7%).

In addition to removing both the bias and the variance, our method also has the advantage of speed. Computation times for the 1, 2, and 7% data shown in this figure were 27, 37, and 70 s (for 1, 2, and 7% missing mass data, respectively) for the conditional logistic approximation versus 4356, 4277, and 4307 s for a naïve summation over all terms. The increased computation time for larger missing masses results from there being more unique patterns. As we show in the next section, the missing mass is experiment specific and is largely a function of firing rates and data length, although population size plays a role as well. Many real neuronal populations, even large ones, spike sparsely and thus have small missing mass. As we will show in the results, in such cases our method cut run times of hours or more down to minutes or seconds.

### 2.3. Computational complexity

Calculation of the stimulus driven missing mass may be split into two steps. (1) Fitting the conditional logistic regression models (step 3 of the above procedure) and (2) Summing terms over each unique pattern observed in the training data (step 4).

Regarding the first step, logistic regression models can be accurately fit by iteratively reweighed least squares methods (Komarek and Moore, 2003; Komarek, 2004) in *O*(*LRF*) time where *L* is the data length (number of time bins), *R* is the number of covariates being regressed upon (the covariate matrix *C*(*s*) is size *L* × *R*) and *F* is the sparsity of the covariate matrix. Here we fit *N* conditioned logistic regression models. For each of these the covariate matrix *C*(*s*) has two components. The first depends upon the stimulus (*F* = 1 except for special cases) and has *R*_stim_ covariates (columns). The second component depends upon the spiking of other neurons, has sparsity *F* and has *n* ∈ 0 … *N* − 1 columns depending upon which model is being fit. Noting that the total sparsity of the covariate matrix with *n* “other neuron” columns is *F*(*n*) = (*R*_stim_ + *Fn*)/(*R*_stim_ + *n*), the total computation time for fitting all *N* logistic regression models is of order

(16) $$\begin{array}{l} L\sum_{n\;=\;0}^{N-1}[F(n)(R_{\text{stim}}+n)]=L\sum_{n\;=\;0}^{N\;-\;1}\left[\frac{R_{\text{stim}}+Fn}{R_{\text{stim}}+n}(R_{\text{stim}}+n)\right]\\ \;=L\sum_{n\;=\;0}^{N\;-\;1}\left(R_{\text{stim}}+Fn\right)\\ \;\approx LN[R_{\text{stim}}+F\frac{N}{2}] \end{array}$$

As the number of neurons *N* grows, this term scales as *O*(LFN^2^/2).

Regarding the second step, this involves summing probabilities over all *N*_pat_ patterns observed in the training data. Each component of this sum requires multiplying *N* − 1 conditional probabilities obtained from the above fitted logistic regression models. Since this is done at all time points there is also a scaling of *L*. Thus the second term scales as *O*(*L*(*N* − 1)*N*_pat_) ≈ *O*(LNN_pat_). The net computational complexity of our algorithm is therefore

(17) $$O({\text{LFN}}^{2}\text{​}/2)+O({\text{LNN}}_{\text{pat}})$$

Since *N*_pat_ > *N* and *F* << 1 (in general) the second term dominates (this is also born out in numerical simulations) and the complexity of our algorithm is roughly *O*(LNN_pat_).

*N*_pat_ is the number of *unique* patterns observed in the data set. This grows with the population size and data length, but at a much slower rate than 2^*N*^. The rate of growth data is data dependent but a rule of thumb estimate can be obtained for a population of Bernoulli neurons each firing with a constant probability *p* per bin. In any time bin, the probability of a pattern with *K* spikes is given by the binomial distribution. *P*_bino_(*N, K, p*). Averaged over the entire data set of length *L*, the number of unique patterns with *K* spikes is approximately upper bounded by:

(18) $$N_{\text{pat}}(K)≲\text{min}\{N!/(K!(N-K)!),P_{\text{bino}}(N,K,p)L\}$$

The first argument, given by the binomial factor, is a hard upper bound, i.e., the total number of possible unique patterns with *K* spikes. The second term, is an approximate upper bound on the total number of times a pattern with *K* spikes is observed in the data set. Its use in the above Equation is conservative, i.e., it is assumed that every time a pattern with *K* spikes is observed it is a *new* pattern. An estimate for the total number of patterns that would be observed in a population of *N* neurons with population mean firing probability *p* over a data set of length *L* is then given by summing the above Equation over all *K* ∈ 0 … *N*. The number of patterns that only occur *once* can be obtained by summing terms over *K* when the binomial probability (second argument) is used for *N*_pat_(*K*), and from this the Good-turing missing mass can be determined.

**Figure 2:**
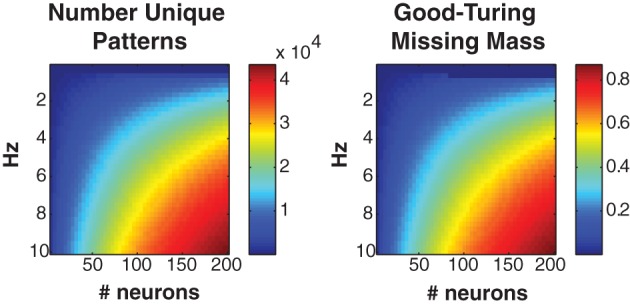
**Missing mass as function of population size and firing rate. Left:** Number of unique patterns observed in a population of Bernoulli neurons with identical firing rates. **Right:** Good-Turing missing mass for same Bernoulli populations. 250 s of data discretized at 5 ms was used. See text for derivation. Note that many neural populations have extremely low <5 Hz mean firing rates, leading to a low number of unique patterns.

Figure 2 shows the number of unique patterns (left) and Good-Turing missing mass (right) for populations of Bernouli neurons with different firing rates (5 ms bins) and neuron numbers. It should be noted that many experimentally recorded neural populations have low mean firing rates (<5 Hz) particularly during naturalistic stimuli (Baddeley et al., 1997; Vinje and Gallant, 2000; Hromadka et al., 2008). The number of patterns will often remain low, even if the population is large. Still, we find empirically that even when higher firing rates are present, the conditional logistic approximation can still attain good results (see section 3.4).

### 2.4. Experimental methods

Data collection for the rat hippocampal population is discussed in Barbieri et al. (2004a,b).

In the case of the macaque DLPFC data (unpublished), procedures were approved by the Massachusetts General Hospital internal review board and were conducted under IACUC-approved guidelines. Anesthesia was induced with Ketamine, Xylazine, and Atropine and maintained with Isoflurane at 2%. Multiple silicone multi-electrode arrays (NeuroNexus Technologies Inc., MI) were surgically implanted in the monkey under stereotactic guidance (David Kopf Instruments, CA). Electrode leads were secured to the skull and attached to female connectors with the aid of titanium miniscrews and dental acrylic. Confirmation of electrode positions was done by direct visual inspection of the sulci and gyral pattern through the craniotomy.

A Plexon multichannel acquisition processor was used to amplify and band-pass filter the neuronal signals (150 Hz 8 kHz; 1 pole low-cut and 3 pole high-cut with 1000x gain; Plexon Inc., TX). Neural signals were then digitized at 40 kHz and processed to extract action potentials by the Plexon workstation. Classification of the waveforms was performed using template matching and principle component analysis based on waveform parameters. Only single-, well-isolated units with identifiable waveform shapes and adequate refractory periods were used. The task involved the presentation of two successive targets on a screen in front of the monkey. After presentation of the targets, the monkeys were given a brief blank screen delay and then a go-cue indicating that they could move, in sequence, to the remembered targets. The monkeys were shown multiple such target sequences over the course of recordings.

The cat data (unpublished) was recorded in Area 18. All experimental procedures were performed in accordance with the Society for Neuroscience and German laws for animal protection and were overseen by a local veterinarian. Anesthesia was initiated by intramuscular injection of ketamine and xylazine and was maintained after tracheotomy by artificial ventilation with a mixture of N_2_O (70%), O_2_ (30%), and halothane (1.2% for surgery and 0.8% for recording) supplemented with intravenous application of a muscle relaxant (pancuronium, 0.25 mg/kg/h) to prevent eye movements. Recording chambers were positioned over the midline at AP2 according to Horseley–Clarke.

The cat was visually stimulated with a black and white high contrast square wave grating of 2.4 cycles per second. The grating was presented pseudo-randomly in one of eight directions: 0, 45, 90, 135, 180, 225, 270, and 315°, respectively. Note that in the results we used only four of these directions (0, 90, 180, and 270) for ease of presentation. Visual stimulation started with showing a gray screen for 2 s followed by one of the four differently oriented gratings remaining stationary for 2 s before the grating started moving for 4 s. Thus, one trial lasted for 8 s. However, here we only consider the 4 s of each trial during which the moving grating was shown (see section 3.4). Spike data was band pass filtered between 800 Hz and 3.5 kHz and then digitized at 20 kHz. Subsequently, action potentials were sorted using template matching procedures for each of the 16 different electrodes.

## 3. Results

We present results for both simulated data and experimentally recorded data sets. For simulated data, we (1) test the conditional logistic approximation in “small” 20 neuron networks, where the true partition function can, with some effort, be calculated and compare with Monte Carlo importance sampling using an independent neuron model proposal distribution, see Appendix B and Bishop (2007); Salakhutdinov (2008). (2) We apply our method to larger networks where the true partition function can not be calculated (up to 90 neurons) and show that it agrees with importance sampling but the result is obtained more quickly and with less variance.

We then apply the method to three experimentally recorded data sets from rat hippocampus, macaque DLPFC and cat Area 18. For “small” (20 neuron) populations we again show that the conditional logistic approximation is more accurate and orders of magnitude faster than importance sampling. For larger populations (41 hippocampal and 39 DLPFC) neurons the results again agree with importance sampling and again are obtained much faster. We also compare to four deterministic approximations [naive mean field, TAP corrected, the Bethe approximation and a low firing rate approximation presented by Roudi et al. (2009a)] and find the conditional logistic approximation to have considerably lower bias and variance than these deterministic approximations.

All computations were performed using Matlab version 7.9.0 R2009B on a single 3.47 GHz core of a Dell Precision T7500 workstation with 48 GB of RAM. Computation times reference the calculation of *Z*(*s*) (over the entire data set) once the Ising parameters are known. Parameters were obtained by fitting stimulus driven Ising models via pseudo-likeihood (see Appendix A), but other methods could be employed and we discuss different possibilities in the Discussion.

### 3.1. Simulated data

**Figure 3:**
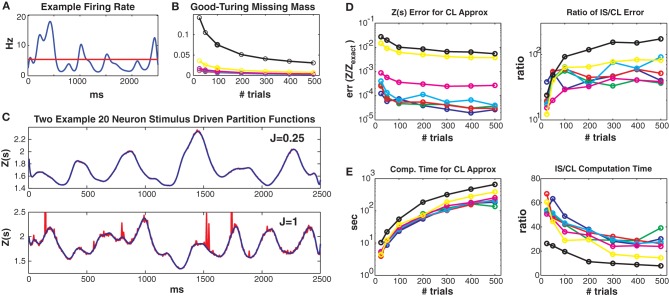
**Simulated 20 neuron populations**. Stimulus driven Ising models with 20 neurons and a repeated trial structure were simulated for various coupling strengths *J*_max_ and numbers of trials (2500 ms long, 5 ms bins). Unless otherwise noted, the values for *J*_max_ = {0.01, 0.05, 0.1, 0.25, 0.5, 1, 1.5} correspond to the colors blue, green, red, cyan, magenta, yellow and black, respectively. See text for further details. **(A)** Time varying firing rate for an example “neuron.” Red line denotes mean firing rate. **(B)** Good-Turing missing masses for different coupling strengths and trial numbers. **(C)** Example *Z*(*s*) for weak (upper) and strong (lower) coupling. Blue is the logistic approximation and red was obtained via importance sampling. *Z*(*s*) calculated by exact summation is in black and indistinguishable by eye from the blue (logistic approximation) line. **(D)** Left: Error of the logistic approximation, defined using the difference between 1 and the 0.5 or 99.5% quantiles of the *Z*_CL_(*s*)/*Z*_exact_(*s*) distribution (see text). Right: Ratio of importance sampling to logistic approximation error. **(E)** Left: Computation time for logistic approximation. Right: Ratio of importance sampling and logistic approximation computation times. For all couplings and trial lengths, the logistic approximation has (1) lower error and (2) faster computation time by at least an order of magnitude.

Simulated data was generated, via Gibbs sampling, using an “protocol” consisting of repeated trials 2500 ms long discretized at 5 ms (500 bins per trial). Stimulus driven Ising models were defined such that each neuron had a firing rate that was strongly variable over each trial but with a 5 Hz mean (see Figure 3A for an example.) Each neuron n's stimulus drive term *h*_*n*_(*t*) was modeled using a linear sum of local (in time) B-spline basis functions *B*_*m*_(*t*) defined on knots spaced at even 100 ms intervals.

(19) $$h_{n}(t)=\sum_{m\;=\;1}^{M}B_{m}(t)\beta_{mn}$$

The *B*_*m*_(*t*) are similar in shape to Gaussians or raised cosines and tile the trial length. The parameters β_*mn*_ control the “height” of these functions. Thus this functional form is roughly equivalent to a smoothed PSTH [see Gerhard et al. (2011) and Haslinger et al. (2012) for further details]. The β_*mn*_ were chosen so that each neuron had roughly a mean 5 Hz firing rate, corresponding to a mean firing probability *p* ≈ 0.025 per bin. For reference, *h*_*n*_(*t*) ∈ [−6, −2] generally. The symmetric coupling matrix *J* was chosen randomly within a range *J* ∈ [−*J*_max_, *J*_max_]. We used seven different values of *J*_max_, i.e., *J*_max_ ∈ {0.01, 0.05, 0.1, 0.25, 0.5, 1, 1.5}. Data was simulated for 9 different sized neural populations: {10, 20, … 90} neurons and 7 different lengths: 25, 50, 100, 200, 300, 400, and 500 trials.

Figure 3 shows results from simulated networks with 20 neurons (small enough so that *Z*(*s*) can be calculated exactly, but large enough so that the computation time is lengthy). Results for all 7 maximum coupling values are shown as different colors (see figure legend). Data lengths are generally plotted along the *x* axes. All these 20 neuron networks had small Good-Turing missing masses, less than 5% except for *J*_max_ = 1.5 (Figure 3B). In Figure 3C we show estimates of *Z*(*s*) via both the conditional logistic approximation (blue) and importance sampling (red) using 5000 MCMC samples for each time bin. The true value for *Z*(*s*) (calculated via exact summation) is in black. The true *Z*(*s*) and conditional logistic approximation are identical by eye. The importance sampling is also identical by eye for weak coupling (*J* = 0.25, upper plot) but visible for the stronger (*J* = 1) coupling in the lower plot. The importance sampling error is larger for stronger coupling because the true Ising distribution is farther from the independent neuron proposal distribution.

Figure 3D left gives the error of *Z*_CL_(*s*)/*Z*_exact_(*s*) for all couplings and data lengths. As described in the methods, we quantify error using the 99% bounds (0.005 and 0.995 quantiles) of the distribution (over stimuli) of this ratio, which is equal to 1 if there is no error. Here, so as to plot a single number, the plotted error is the maximum of the difference between 1 and either the 0.005 or 0.995 quantile of the ratio distribution. The error is small for all simulations, but largest for the highest coupling levels because stronger coupling results in more unique patterns and larger missing masses. However, as we show in Figure 3D right, the error is always smaller than that of importance sampling. Here we show the ratio of the *Z*_*IS*_(*s*) error to that of *Z*_CL_(*s*). The importance sampling error is larger by 1 to 2 orders of magnitude. Of course this ratio will go down if more MCMC samples are used, but that requires more computation time. Figure 3E left gives the computation time for all coupling strengths and trials. Computation time increases with both coupling strength and data length (although sub-linearly) because there are more unique patterns. However, as shown in Figure 3E right, our conditional logistic approximation is always faster (by 10–60 times) than importance sampling.

**Figure 4:**
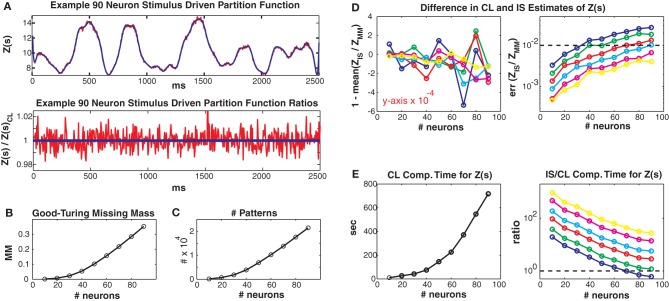
**Larger simulated populations**. Unless otherwise noted, *J*_max_ = 0.25 and 100 trials are used. **(A)** Upper: *Z(s)* for an 90 neuron population. Blue: logistic approximation, red: importance sampling. Lower: ratio of importance sampling over logistic approximation (red). Blue line is equal to 1 and indicates equality of the two methods. **(B)** Good-Turing missing mass and **(C)** number of unique patterns, for various population sizes. Note that the 5 Hz population mean firing rate is somewhat high for real data. **(D)** Left: Mean bias of ratio between importance sampling and logistic approximations for *Z(s)*. Right: Error of partition function ratio. Colors refer to different numbers of MCMC samples used in importance sampling, i.e., blue, green, red, cyan, magenta and yellow refer to 1000, 2000, 5000, 10,000, 25,000 and 50,000 samples *per time bin*. Horizontal dashed line equals 0.01 and indicates a 1% error for the importance sampling method. **(E)** Left: Computation time for logistic approximation. Right: Ratio of importance sampling computation time to logistic computation time. Horizontal dashed line equals 1. Note that for all simulations where the importance sampling error was less than 1% the computation time for the importance sampling was slower than for the conditional logistic approximation.

In Figure 4 we consider larger populations where it is not possible to exactly calculate the stimulus driven partition function. Instead, we compare our conditional logistic approximation to importance sampling for different numbers of MCMC samples (1000, 2000, 5000, 10,000, 25,000, 50,000) per data point, denoted by colors (see figure caption) and for different numbers of neurons (generally along *x* axes) ranging from 10 to 90. We use *J*_max_ = 0.25 and 100 trials but results for other coupling strengths and data lengths are qualitatively similar. Figure 4A upper shows the conditional logistic estimated *Z*(*s*) in blue and the importance sampling estimate (5000 MCMC samples) in red for an 80 neuron population. Figure 4A lower shows the difference between the importance sampling and conditional logistic approximations.

**Figure 5:**
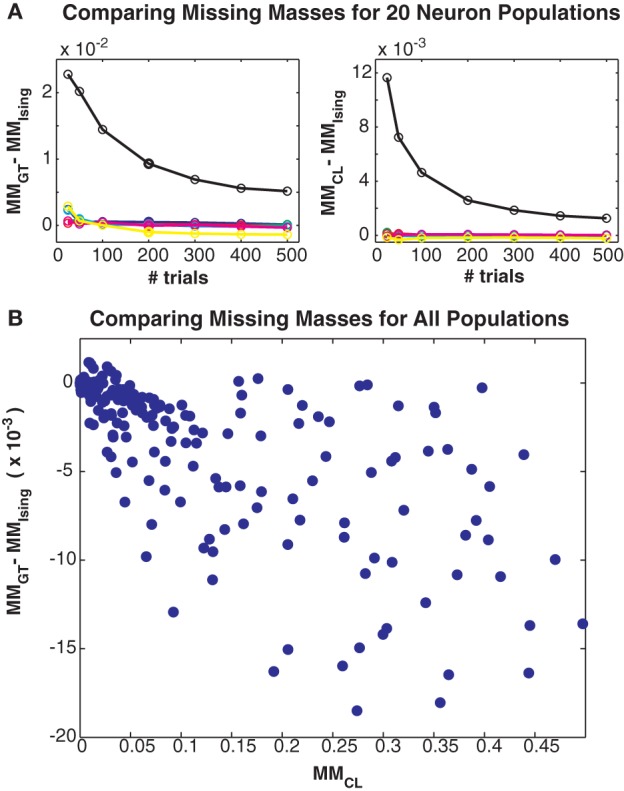
**Comparing different missing mass estimates. (A)** Left: Difference between Good-Turing and exact summation stimulus averaged missing masses for all 20 neuron simulations. Colors correspond to maximum coupling strengths as in Figure 3. Right: Difference between conditional logistic and exact summation missing masses. **(B)** Scatter plot of difference between Good-Turing and logistic approximation missing masses versus the conditional logistic missing mass. Note that this plot includes results from large populations so exact summation is not possible. All three estimates closely agree, suggesting that the Good-Turing estimate is roughly unbiased for neuronal data.

**Figure 6:**
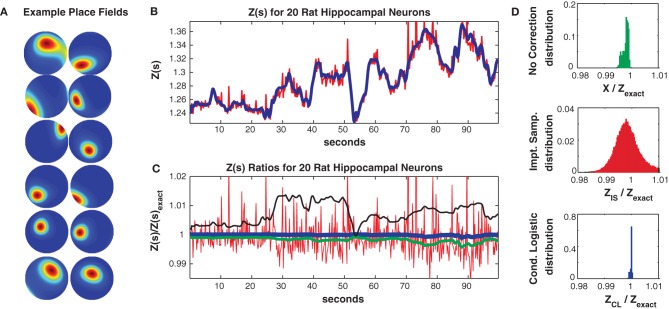
**Twenty neuron population from rat hippocampus. (A)** Ten representative single neuron place fields. **(B)** *Z(s)* for logistic approximation (blue) and importance sampling (red). Exact value is in black and indistinguishable by eye from blue. **(C)** Ratios of various approximations for *Z(s)* with respect to exact value. Blue: Logistic approximation, red: importance sampling, green: *X(s)* e.g., no correction, black: independent neuron model. **(D)** Error distributions for: upper: no correction *(X(s))*, middle: importance sampling, lower: logistic approximation. The logistic approximation removes the bias and the variance and is calculated in seconds (see text for computation times).

**Figure 7:**
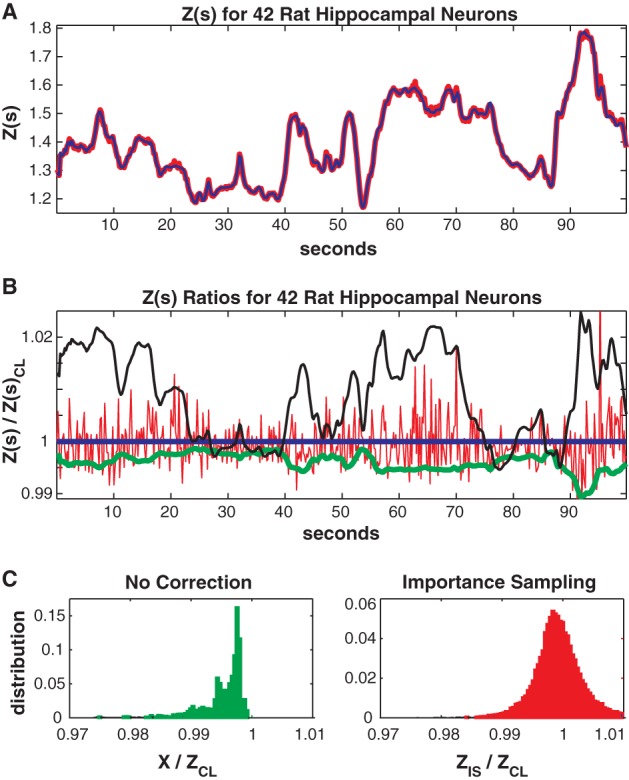
**Forty-two neuron population from rat hippocampus. (A)** *Z(s)* for logistic approximation (blue) and importance sampling (red). **(B)** Ratios of various approximations for *Z(s)* with respect to logistic approximation. Blue: Logistic approximation (equal to 1 by definition). Red: importance sampling, green: *X(s)* e.g., no correction, black: independent neuron model. **(C)** Error distributions for: left: no correction *(X(s))*, right: importance sampling. The importance sampling result has the same average bias as the logistic approximation but is noisier. Moreover the logistic approximation is much faster to calculate (see text).

**Figure 8:**
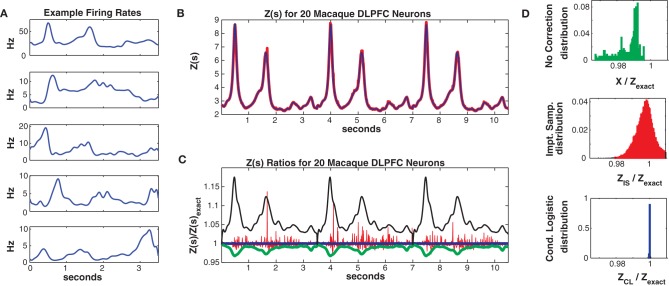
**Twenty neuron population from macaque DLPFC. (A)** Five representative single neuron firing rates. **(B)** *Z(s)* for logistic approximation (blue) and importance sampling (red). Exact value is in black and indistinguishable by eye from blue. **(C)** Ratios of various approximations for *Z(s)* with respect to exact value. Blue: Logistic approximation, red: importance sampling, green: *X(s)* e.g., no correction, black: independent neuron model. **(D)** Error distributions for: upper: no correction *(X(s))*, middle: importance sampling, lower: logistic approximation. The logistic approximation removes the bias and the variance and is calculated more quickly (see text).

The two methods agree very well even though they started from different “null” distributions. That is, the conditional logistic approximation started using *X*(*s*) (calculated using only the patterns observed in the training data) which is by definition *less than Z*(*s*). In contrast, the importance sampling started from an independent neuron proposal distribution which had a *higher* estimate of *Z*(*s*) than the final importance sampling result (independent neuron approximation for *Z*(*s*) not shown because outside of plot range but see Figures 5–8 for examples). The fact that the two approximation methods converge onto the same answer, despite their different starting points lends confidence that both methods give un-biased estimates. However, the importance sampling estimate is much *noisier*, distributed around our conditional logistic approximation. In Figure 4B we show how the Good-Turing missing mass changes as a function of neuron number. The increase in pattern number for larger populations is due to the relatively high population mean firing rate (5 Hz) (Figure 4C). We emphasize that many experimental data sets (see below) have population mean firing rates less than 5 Hz.

We next compare to importance sampling for different numbers (ranging from 1000 to 50,000) of MCMC samples per time bin. Figure 4D left shows the mean, over the entire data set, of the difference between 1 and *Z*_*IS*_(*s*)/*Z*_CL_(*s*). This difference is small, usually on the order of 10^−4^ regardless of neuron or MCMC sample number. This indicates that the bias of the two methods agrees for all simulations. However, the error (99% bounds) of *Z*_*IS*_(*s*)/*Z*_CL_(*s*) is always larger than this mean, indicating that importance sampling is always noisier, even for large numbers of MCMC samples. As more MCMC samples are used, the error decreases and the two methods converge indicating that our conditional logistic approximation is accurate. Notably, as the population size grows, more MCMC samples are required to obtain as accurate a fit as our conditional logistic approximation. The dashed line indicates an error of 0.01 (1%). Moreover, the conditional logistic approximation is always *faster* if enough MCMC samples are used to obtain an accurate estimate of *Z(s)*. Figure 4E (left) gives the conditional logistic computation time and Figure 4E right gives the ratio of the importance sampling to conditional logistic computation times. This ratio is always on the order of 10 or higher if enough MCMC samples are used to have an error less than 1%.

Finally, in Figure 5 we compare the Good-Turing estimate of the missing mass with the missing mass as estimated from the Ising model and via our conditional logistic regression approach. Figure 5A left shows the difference between the Good-Turing and Ising estimates for all 20 neuron populations, while Figure 5A right shows the difference between the conditional logistic and Ising estimates. When averaged over all stimuli, our conditional logistic approximation for the missing mass agrees very well with the exact Ising missing mass for all models. Further, while slightly less accurate, the Good-Turing estimate is also very good, particularly when the missing mass is low. Figure 5B compares the Good-Turing and conditional logistic estimates for all simulations (where for the larger populations, the Ising missing mass can not be determined exactly). Again, the estimates agree very well lending confidence that the Good-Turing missing mass is a good, and fast, approximation for the stimulus averaged missing mass.

In summary, we tested our missing mass approximation for a range of population sizes, data lengths and coupling strengths and compared it to importance sampling using different numbers of MCMC samples. In all cases the missing mass approximation was more accurate, and took less computation time if enough MCMC samples were used to obtain low error. It is possible that a different importance sampling proposal distribution (perhaps based upon Gibbs sampling using the Ising model parameters) would produce more accurate importance sampling estimates. However, then computation time would drastically increase. For reference, the Gibbs sampler we used to generate the simulated data took 10/39 s to produce to produce 5000 samples for a 20/90 neuron population while the independent neuron sampler took 0.02/0.04 s. Note that 5000 or more samples are required *per time bin*.

### 3.2. Experimental data

We now demonstrate our method using 3 different data sets: 41 rat hippocampal neurons, 39 macaque DLPFC neurons and 20 cat Area 18 neurons. The hippocampal data was recorded as a rat explored a circular maze, the DLFPC data was recorded as a monkey performed an associative memory task involving repeated stimulus presentations over trials and trials and the anesthetized cat data was recorded as a cat was stimulated with 4 different stimuli consisting of high contrast gratings moving at 4 different orientations (0, 90, 180, and 270°). As with our simulated results, stimulus driven Ising models were fit via pseudo likelihood prior to calculating *Z*(*s*).

#### 3.2.1. Rat hippocampus

We used 41 place cells recorded from rat hippocampus as a rat explored a circular maze. This data is the same as used in Barbieri et al. (2004a,b) which has discussions of the experiment. 1000 s of data was used, discretized into 10 ms bins and split into 75% training and 25% test sets. Place cells code the rat's position in space, i.e., the circular enclosure, by firing strongly when the rat is in a specific physical location called the cell's “place field.” Here we parameterized the rat's location (stimulus) using a linear sum of the first 10 Zernike polynomials (Barbieri et al., 2004a). Zernike polynomials constitute a set of complete basis functions on the unit disc. Each neuron's stimulus drive term was therefore modeled as:

(20) $$h_{n}(t)=\sum_{m\;=\;1}^{10}\zeta_{m}(\rho (t),\theta (t))\beta_{mn};$$

where ζ_*m*_(ρ(*t*), θ(*t*)) is the m'th Zernike polynomial which is a function of the rat's position (stimulus) in polar coordinates *s*(*t*) = {ρ(*t*), θ(*t*)} and β_*nm*_ are fitted parameters. The mean firing rate of this population was low (0.8 Hz) but neurons were selective for the rat's location and fired strongly in their place fields (mean maximum firing rate 8.6 Hz). Figure 6A shows ten example single neuron place fields obtained by fitting single neuron logistic regression models (Gerhard et al., 2011; Haslinger et al., 2012) with the above stimulus covariate matrix.

In Figure 6 we consider a subpopulation of 20 neurons (those with the highest mean firing rates) so that *Z*(*s*) can be calculated by exact summation. This subpopulation exhibited 321/230 unique patterns in the training/test data of which 113/98 appeared only once. The Good Turing missing mass (calculated from the training data) was 0.0015. In comparison the missing mass calculated by fitting a stimulus independent Ising model was 0.0018 and the estimate obtained by averaging the conditional logistic missing mass was 0.0018. Figure 6B compares *Z*(*s*) over a 100 s epoch determined via exact summation (black), importance sampling (red) and the conditional logistic approximation (blue). The black and blue lines are identical by eye. In Figure 6C we show the ratio (with respect to *Z*(*s*)'s exact value) of the importance sampling result for *Z*(*s*) (red) and conditional logistic approximation (blue). We also show analogous ratios for *X*(*s*) (no correction) in green and the partition function calculated from the independent neuron model used as the importance sampling proposal distribution (black). Figure 6D shows the distribution of these ratios over the entire test data set for no correction (upper, green), importance sampling (middle, red), and the conditional logistic approximation (lower, blue). The independent neuron distribution is not shown because it is outside the range of the plots. Our method is extremely accurate, the mean of the error distribution is 0.9999 and the 99% quantiles of the distribution is {0.9989, 1.0002}. In contrast, the importance sampling confidence bounds are {0.9878, 1.0451} although it is also unbiased (mean = 0.9998).

Crucially, however, the computation time was much faster for the conditional logistic approximation. It took 8.1 s to evaluate *Z*(*s*) for the training data and 7.1 s for the test data. In contrast, exact summation took 1928 and 607 s for training and test data, respectively while importance sampling took 1531 and 513 s for training and test data, respectively.

In Figure 7 we consider the full 41 neuron population. The training/test data had 817/551 unique patterns of which 386/113 patterns occurred only once. The Good-Turing missing mass was 0.0051 while the result from our conditional logistic approximation was 0.0049. Figure 7A shows *Z*(*s*) for both importance sampling (red) and our method (blue). Since exact summation is not feasible, Figure 7B shows ratios *with respect to the conditional logistic approximation* for *Z*(*s*). The blue line is therefore the missing mass divided by itself, equal to 1 by definition. Red is the ratio of importance sampling result over the missing mass approximation, green *X*(*s*) (no correction) and black the independent neuron approximation. Ratio distributions, over all test data stimuli, are shown in Figure 7C. The importance sampling distribution has mean 0.9999, indicating that importance sampling and the missing mass approximation have the same bias. Given that the two methods had different starting points (green and black lines) the fact that their means agree so well suggests that they are converging on the correct answer. However, the importance sampling result is noisier, with 99% confidence bounds of {0.986, 1.027}. Furthermore, the missing mass approximation is again faster: 46/36 s for training/test data while the importance sampling result was 1801/605 s, respectively. Exact summation results are not possible.

### 3.3. Macaque DLFPC

We used a 39 neuron population recorded in Macaque DLFPC as the monkey performed an associative memory task where it viewed two targets in succession and then moved a joystick to those targets. This task was repeated over 300 separate trials, each 3500 ms long. (Unpublished data, see Experimental Methods and also Pellizzer et al. (1995) for a similar task structure). Here, dynamic changes in the network function are produced by the task structure, i.e., target, delay, second target, movement. To parameterize this structure we used the time since trial onset as the stimulus, similar to our simulated data. Thus the time varying drive to each neuron was again parameterized using a sum of 4th order B-spline basis functions which tiled each trial. That is:

(21) $$h_{n}(t)=\sum_{m\;=\;1}^{38}B_{m}(t)\beta_{nm}$$

Spline knots were spaced 100 ms apart, resulting in 38 localized (in time) basis spline functions *B*_*m*_(*t*). The β_*nm*_ are fitted parameters. The data was discretized into 10 ms bins (350 per trial) and was again partitioned into 75% (225 trials) training and 25% (75 trials) test sets. The population mean firing rate was 2.1 Hz but again individual neuron firing rates varied strongly, here as a function of time since trial onset with a mean maximum firing rate (across the population) of 7.2 Hz. Figure 8A shows 5 example individual neuron firing rates.

In Figure 8 we consider a sub-population of 20 neurons with the highest firing rates. Training/test data had a 2011/1079 unique patterns, 1014/573 of which occurred once. The Good-Turing missing mass was 0.013 while the Ising missing mass was 0.013 and the mean conditional logistic missing mass was also 0.013. Figure 8B compares *Z*(*s*) over 3 trials (10.5 s) as determined via exact summation (black), importance sampling (red) and the conditional logistic approximation (blue). The black and blue lines are again identical by eye. In Figure 8C we show the ratio, with respect to *Z*_exact_(*s*), for importance sampling (red) and the conditional logistic approximation (blue). We also show analogous ratios for *X*(*s*) (no correction) in green and for the independent neuron approximation used as the importance sampling proposal distribution (black). Figure 8D shows the distribution of these ratios over the entire test data set for *X*(*s*) (upper, green), importance sampling (middle, red), and the conditional logistic approximation (lower, blue). The independent neuron distribution is not shown because it is outside the range of the plots. Our method is extremely accurate, the mean of the ratio distribution is 0.9999 and the 99% quantiles are {0.9992, 1.0003}. In contrast, the importance sampling confidence bounds are {0.9818, 1.0408} although it is also unbiased (mean = 0.9999).

Again, the computation time was much faster for the conditional logistic approximation. It took 34 s to evaluate *Z(s)* for the training data and 21 s for the test data. In contrast, exact summation took 2020 and 720 s for training and test data, respectively while importance sampling took 1621 and 548 s for training and test data, respectively.

**Figure 9:**
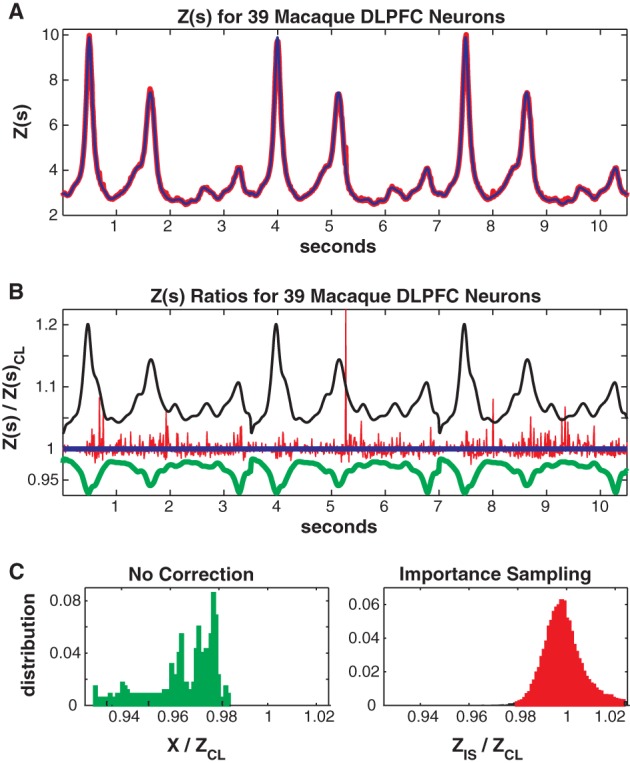
**Thirty-nine neuron population from macaque DLPFC. (A)** *Z(s)* for logistic approximation (blue) and importance sampling (red). **(B)** Ratios of various approximations for *Z(s)* with respect to logistic approximation. Blue: logistic approximation (equal to 1 by definition). Red: importance sampling, green: *X(s)* e.g., no correction, black: independent neuron model. **(C)** Error distributions for: left : no correction *(X(s))*, right: importance sampling.

In Figure 9 we consider the full 39 neuron population. Here the training/test data had 4452/2173 unique patterns 2705/1377 of which occurred once. The Good-Turing missing mass was 0.034 and the mean conditional logistic missing mass was also 0.034. Figure 9A shows *Z*(*s*) for both importance sampling (red) and the conditional logistic approximation (blue). Since exact summation is not feasible, Figure 9B show's ratios *with respect to the missing mass approximation* for *Z*(*s*). The blue line is therefore the missing mass divided by itself, equal to 1 by definition. The red line is the ratio of the importance sampling result *Z*_*IS*_(*s*) over *Z*_CL_(*s*), green *X*(*s*) (no correction) over *Z*_CL_(*s*) and black the independent neuron approximation over *Z*_CL_(*s*). Distributions of these ratios, over all test data stimuli, are shown in Figure 9C. The importance sampling distribution has mean 1.0001, indicating that importance sampling and the missing mass approximation have the same bias. Given that the two methods had different starting points (green and black lines) the fact that their means agree so well suggests that they are converging on the correct answer. However, the importance sampling result is noisier, with 99% confidence bounds of {0.9805, 1.0522}. Furthermore, the conditional logistic approximation is again faster: 111/66 s for training/test data while the importance sampling result was 1944/664 s, respectively.

### 3.4. Cat area 18

Finally we present an example using 20 neurons recorded in Area 18 of an anesthetized cat as a high contrast grating was shown for 4 s in one of 4 different (90° rotated) directions (unpublished data see Experimental Methods). 21 training trials from each direction (84 total) were used and 7 test trials (28 total). Area 18 neurons are known to be highly direction dependent, so the time varying drive to each neuron was allowed to vary both as a function of direction and time since stimulus onset. Specifically, as with the macaque data, we used basis spline expansions (200 ms knot spacing) as a the function of time since stimulus onset as in Equation 2.1. However, the splines were different in each of the 4 directions allowing for directional tuning.

**Figure 10:**
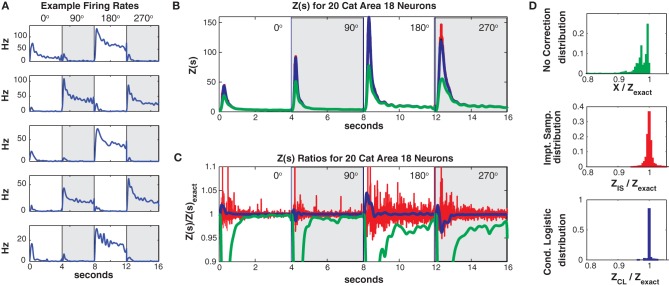
**Twenty neuron population from cat Area 18. (A)** Five representative single neuron firing rates. Different background shadings denotes responses from different (90° rotated) grating directions. Thus the onsets of the different grating directions occur at 0, 4, 8, and 12 s, respectively. Note that this is only for plotting purposes, trials were separated by a delay in the actual experiment (see text). **(B)** *Z(s)* for logistic approximation (blue), importance sampling (red) and no correction (green). Exact value is in black and indistinguishable by eye from blue. **(C)** Ratios of various approximations for *Z(s)* with respect to exact value. Blue: logistic approximation, red: importance sampling, green: *X(s)* e.g., no correction. The independent neuron model is not shown because it is outside the plot. **(D)** Error distributions for: upper: no correction *(X(s))*, middle: importance sampling, lower: logistic approximation.

Data was discretized into 5 ms bins (800 per trial and direction). For this data set the mean population firing rate was much higher (21.4 Hz, highest neuron firing rate = 47 Hz) than in the two previous examples. As can be seen in Figure 10A, the individual neurons firing rates were strongly direction tuned and also had a strong “on” response at the onset of the grating stimulus. The high firing rates led to a larger number of unique patterns. The training/test data had 7018/2013 unique patterns (4547/1422 of which occurred once) and a higher Good Turing missing mass of 0.071 (conditional logistic missing mass of 0.068) than in our previous examples.

Despite the larger missing mass, the conditional logistic approximation performed very well as can be seen in Figures 10B,C which show *Z*(*s*) and the ratio of *Z*(*s*)/*Z*_exact_(*s*), respectively. The 99% confidence bounds on the error (Figure 10D) were somewhat larger {0.9654, 1.0405} than in our previous examples. This error appeared to be localized to the peak of the partition function at stimulus onset, away from the peak the error was quite small. The peak error resulted in “tails” in the error distribution which contained a relatively small proportion of the distribution (Figure 10D bottom). e.g., the 90% bounds on the distribution were within a percent {0.9964, 1.0083} and the distribution itself was unbiased (mean = 1.0003). Moreover, these results should be compared to the error with no correction (99% quantiles {0.4449, 0.9996}, 90% bounds {0.6376, 0.9973}) the importance sampling error (99% bounds {0.9651, 1.0598}, 90% bounds {0.9848, 1.0153}). Again the conditional logistic approximation was quickest (in addition to being the most accurate) 80/52 s for training/test data compared to 1407/486 s for importance sampling and 1636/588 for naive summation.

### 3.5. Comparison with deterministic approximations

In addition to importance sampling, numerous deterministic approximations to the partition function exist (Opper and Saad, 2001). These often provide a lower bound upon the partition function which, as we demonstrate in this section, this can lead to highly biased error distributions for *Z*(*s*)/*Z*_exact_(*s*) and overestimated pattern probabilities. We compared our conditional logistic approximation to four deterministic approximations of the partition function: (1) Naive mean field theory, (2) TAP corrected mean field theory, (3) the Bethe approximation fit via loopy belief propagation, and (4) a “low firing rate” approximation presented by Roudi et al. (2009a). We describe each of these approximations and give the main results necessary to apply them in Appendix C. We do not provide computation times because these methods are almost instantaneous (second or less) to apply.

**Figure 11:**
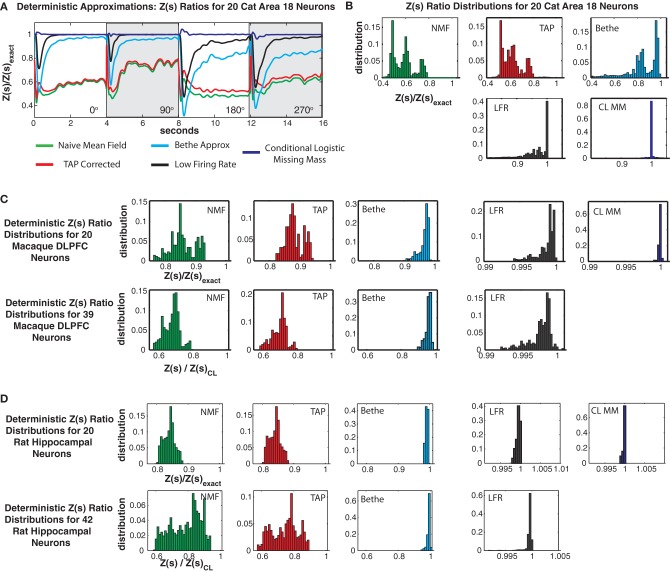
**Comparison with deterministic approximations. (A)** Cat Area 18 partition function ratios for naive mean field theory (NMF, green), TAP corrected mean field theory (TAP, red), Bethe approximation (Bethe, cyan), low firing rate approximation (LFR, black) and the conditional logistic approximation (CL MM blue). **(B)** Error distributions for the different approximations. Note that all deterministic approximations exhibit not only large variance but also a strong negative bias. Also note that while the NFM, TAP and Bethe distributions are plotted on the same scale, the scale for the LFR and CL MM distributions is smaller. **(C)** Error distributions for Macaque DLPFC neurons. **(D)** Error distributions for rat hippocampal neurons.

Figures 11A,B shows *Z*(*s*) ratios and error distributions for the four deterministic approximations applied to the cat Area 18 data and compares the results to the conditional logistic approximation. All four approaches severely under-estimate the partition function. Naive mean field theory performs the worst, followed by TAP corrected and the Bethe approximation. Moreover, these variational type approaches produce estimates of *Z*(*s*) with high bias and variance, a result which also holds for the monkey and rat data sets (Figures 11C–D).

Roudi's low firing rate approximation proved to be the best of the deterministic approximations we considered. Yet it still had a larger bias and variance than the conditional logistic approximation. This was most acute for the cat data, where it produced 99% error bounds of {0.4927, 0.9999} (90% bounds of {0.7124, 0.99951}) and a mean of 0.9404. This should be compared to the 99% conditional logistic bounds of {0.9654, 1.0405} (90% {0.9964, 1.0083}) and the low bias mean (1.0003) of our conditional logistic approximation. It should be noted that Roudi and colleagues explicitly state in Roudi et al. (2009a) that theirs is a low firing rate approximation. Hence it is not surprising, that it performs poorly for the cat data which has a population mean firing rate of 21.4 Hz. In the case of the rat data (Figure 11D), which has very low firing rates, Roudi's approximation performs extremely well. However, in all cases, our conditional logistic approximation provides a more accurate (smaller bias and variance) estimate of the partition function.

## 4. Discussion

The Ising model has gained popularity as a way to describe population spiking in part because it describes the population's second order structure (firing rates and pair-wise correlations between neurons) without making any further assumptions. That is, it is the maximum entropy (most disordered) distribution under these second order constraints (Jaynes, 1957; Roudi et al., 2009b). However, the Ising model does pose some computational challenges arising from the couplings between neurons being undirected and instantaneous. This means there is no closed form which will normalize the probability distribution, and therefore that normalization has to be accomplished via explicit summation or some approximate method. In part for this reason, Ising models generally have not included stimulus drive (Martignon et al., 2000; Schneidman et al., 2006; Tang et al., 2008; Roudi et al., 2009b; Ganmor et al., 2011) (but also see below). Static, non-stimulus-dependent, Ising models, for which the partition function is constant over the data set, are difficult enough to evaluate even when Monte Carlo methods are used. If stimulus drive is included, the partition function can potentially be different in every single time bin. However, to study population coding of stimuli, such drive must be included.

Here we presented a method to quickly (within minutes or less) calculate the partition function for a stimulus driven Ising model. This relied upon the fact that most real neural populations spike sparsely and hence most possible patterns are extremely improbable. Thus we only explicitly summed terms corresponding to patterns which appeared in training data and recast the remainder of the sum in terms of the missing mass. We showed that for stimulus independent Ising models the missing mass can be approximated using the Good-Turing estimator, which relies upon counting patterns (Good, 1953; Orlitsky et al., 2003), while for stimulus driven Ising models a product of conditional logistic regression models can be used. We found this conditional logistic approximation to be more accurate than both deterministic (variational) methods and Monte Carlo importance sampling.

The partition function is central to statistical mechanics and machine learning and many techniques for approximating it have been developed. These generally fall into two classes: stochastic and deterministic. Stochastic methods, such as importance sampling, tend to be slow but converge reliably to unbiased estimates in the limit of large sample size. Using both simulated data and 3 experimentally recorded data sets we showed that our method can calculate the partition function more accurately than Monte Carlo based importance sampling and can do so orders of magnitude more rapidly. Deterministic approximations such as mean field theories, variational methods, and perturbative expansions are extremely fast, but provide lower bounds on the partition function which can have large bias. We compared our missing mass approximation to four deterministic approximations: (1) Naive mean field theory, (2) TAP corrected mean field theory, (3) the Bethe approximation fit via loopy belief propagation, and (4) a “low firing rate” approximation presented by Roudi et al. (2009a,b). For all three experimental data sets, these deterministic approaches produced (at times very) biased results with higher variance than our conditional logistic approximation.

The Ising model has traditionally been used to study magnetism on crystal lattices. It was initially proposed by Lenz (1920) and the one dimensional case was solved by his student Ernst Ising (Ising, 1925). The two dimensional case was solved much later by Onsager (1944). A good history can be found in Brush (1967). For magnetism, undirected and instantaneous couplings make sense, electronic spins do interact in a symmetric and instantaneous manner. Moreover, the regularity of the lattice makes it clear which atoms interact, neighbors and next nearest neighbors. Also translational and rotational symmetries make mean field methods highly applicable (Opper and Saad, 2001; Nguyen and Berg, 2012). These considerations make the problem easier in some respects, and many methods for solving the Ising model rely upon them (Kotze, 2008; Friel et al., 2009). For example, many methods improve partition function estimation by utilizing structure in the connectivity matrix to “cluster” tightly connected spins (Cocco and Monasson, 2011).

In the case of neurons it is not *a priori* clear which neurons are interacting, and these interactions are fundamentally directed, by synaptic contact, and time lagged, by the time it takes an action potential to propagate down an axon. Still, if one is interested in correlations between neurons at time scales of ~5–10 ms then the Ising model is a very useful statistical framework. It has been used to demonstrate the existence of second and also higher order correlations between neurons (Martignon et al., 2000; Schneidman et al., 2006; Tang et al., 2008; Roudi et al., 2009b; Ganmor et al., 2011). Such studies have conclusively shown that the activity of many neuronal populations is *collective* and that neurons can often not be considered as independent coders. Ising models have also shown that consideration of correlations can sometimes improve decoders, demonstrating that correlations may carry useful information (Schaub and Schultz, 2008). Recently, several groups have begun to include time varying stimulus drive (Tkacik et al., 2010; Granot-Atedgi et al., 2013). Such efforts are crucial because correlations between neurons are weak, and most commonly stimuli and neurons' own auto structure explain a much greater fraction of the population spiking statistics. We note that the couplings themselves may also be stimulus modulated. Such modulations are, however, difficult to detect due to the sparsity of coincidences (between neurons) in neural spiking. Developing methods for studying stimulus modulated correlations is an active field of research (Haslinger et al., 2013).

In order to efficiently fit Ising models, either static or stimulus driven, it is necessary to use methods that do not require explicit partition function calculation. Several techniques which use gradient information to maximize the likelihood (or equivalently minimize the Kullback Leibler divergence) without calculating the partition function have been developed. Monte Carlo techniques rely upon the fact that gradients, with respect to the parameters being fit, can be estimated by calculating expectations with respect to the Ising model distribution (Tkacik et al., 2006; Bishop, 2007; Broderick et al., 2007). Since expectations are integrals over a probability distribution, they can be approximated by Monte Carlo sampling from that distribution and summing. Mean field methods have been used to perform parameter estimation (Mezard and Mora, 2009; Roudi et al., 2009a,b; Nguyen and Berg, 2012) although some authors claim them to be inferior to methods such as pseudo likelihood and minimum probability flow, at least for certain data sets (Sohl-Dickstein et al., 2011). Minimum probability flow establishes deterministic dynamics on the state space of all patterns and uses coordinate descent based on these dynamics to fit the Ising model without sampling or partition function calculation. It is extremely fast and can also be used for models defined on continuous state spaces (Sohl-Dickstein and Culpepper, 2012). A third technique, which we used in this paper, is pseudo-likelihood which determines Ising parameters by fitting the Ising conditional probabilities which are exactly logistic regression models. All these methods can be extended to include stimulus drive.

We emphasize that our missing mass approach for calculating the partition function does not depend upon the exact method used to previously fit the Ising model. We chose to use pseudo-likelihood because (1) logistic regression models are fast to fit if conjugate gradient methods are used (Komarek and Moore, 2003) and (2) logistic regression has long been used in the context of Generalized Linear Models (GLMs) to fit neuronal population data so the machinery of how to include stimuli (and spike history if need be) is well developed (Truccolo et al., 2005). Toward this later point, the Ising conditional probabilities (logistic regression models) fit in the pseudo-likelihood approach are in fact GLMs with logit link functions. Thus any effect (stimulus, population spike history, LFP, etc.) which can be included in a GLM can also be included in a pseudo-likelihood fit Ising model by subsuming it into the time varying fields $\vec{h}(s)$.

Another advantage of pseudo-likelihood which we did not pursue here, is that it lends itself to fitting sparse (in the interactions) models. Because the neurons are fit independently (but conditioned on each other), the same L1 regularization (Schmidt et al., 2007; Pillow et al., 2008) or *p*-value (Gerhard et al., 2011) based variable selection techniques that have long been applied to GLM inference of functional interactions between neurons (Pillow et al., 2008; Gerhard et al., 2011) can also be applied here. This was done in Aurell and Ekeberg (2012) and indeed logistic regression has long been known to be effective for Markov random field edge detection (Schmidt et al., 2007). We also note that pseudo-likelihood could be used as a *initial condition* for either Monte Carlo, or minimum probability flow methods. Regardless of how stimulus driven Ising models are fit, they must always be normalized, and that is what we focused on in this paper.

Normalization is a necessary step for any model which is a Markov random field, that is, can be represented as an undirected graph. It is not required if the model can be represented as a directed graph. As an example, an directed graph approach which has found great application for analyzing neuronal populations is the Generalized Linear Model (GLM) method (Truccolo et al., 2005; Pillow et al., 2008; Gerhard et al., 2011) and also see Tyrarcha et al. (2013). Here, each neuron's probability of spiking is conditioned upon the *past* spiking of all other neurons in the population. Causality allows the conditioning to be “one way”, i.e., a spike at time *t* is conditioned on the spikes at time *t*′ < *t* but not vice versa. Hence GLMs can be represented by directed graphs and each neuron can be fit individually, but conditioned upon the other neurons' past spiking histories. However, in order for the conditional independence assumption to hold, the time bins must be taken to be small on the order of a millisecond. This insures that there is no dependence between neurons in the same time bin.

In essence, our conditional logistic approximation uses a directed graph model to approximate the probabilities of all patterns not observed in the training data. The assumption is that since these probabilities are small, errors will roughly average out when they are summed over all missing mass patterns. The directed graph model is implicitly defined through the product of *N* subset conditioned logistic regression models and this model is itself normalized over all patterns. However, it is a slightly different model than the true stimulus driven Ising model. and the probabilities of the two models are not exactly identical. This lack of equivalence arises because the marginals of the Ising model (subset conditioned probabilities) do not have the same functional form as the fully conditioned probabilities and are not exactly logistic regression models, although the fully conditioned probabilities are. Formally this property that the marginals do not have the same form as the true conditionals means that the Ising model is not *projective* (Shalizi and Rinaldo, 2012). What we have shown is that for sparsely spiking networks, the use of logistic regression for the subset conditioned probabilities makes stimulus driven Ising models *approximately* projective. Moreover our conditional logistic approximation is extremely accurate (especially when compared to mean field theories) and extremely fast (when compared to importance sampling or naive summation).

The advantages of our approach are speed and by extension, the ability to quickly calculate *Z(s)* for larger populations. For our method, speed is primarily a function of the number of unique patterns in the data (sparsity), rather than the population size. When combined with a fast method for estimating the model parameters (pseudo likelihood or minimum probability flow) the conditional logistic approximation allows Ising models to be efficiently used for studying population coding in larger populations as long as they spike sparsely. Fortunately, this is the case for many neuronal populations, at least under naturalistic conditions. Fundamentally then, our method allows Ising models to be used to investigate the dynamic *function* of networks rather than only their static structure.

### Conflict of interest statement

The authors declare that the research was conducted in the absence of any commercial or financial relationships that could be construed as a potential conflict of interest.

## Figures and Tables

**Figure A1:**
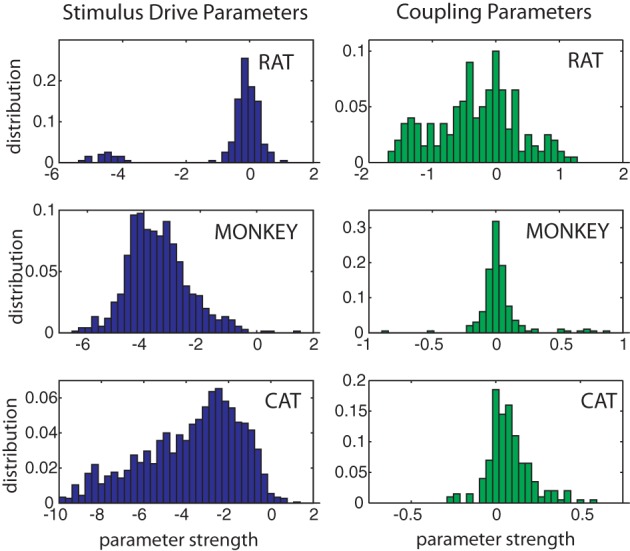
**Examples of pseudo-likelihood fit parameters**. Left column shows histograms of stimulus drive parameters β_*nm*_ the right shows histograms of parameters of the coupling matrix *J*. Results from 20 neuron rat, monkey and cat data are shown. See Appendix A for discussion.

## Appendices

### Appendix A: fitting driven ising models via pseudolikelihood

Pseudolikelihood techniques obtain the parameters of a joint distribution (such as the Ising model) by fitting a set of conditional distributions (Besag, 1974, 1975). In the case of the Ising model (either stimulus driven or not) the conditional distributions are exactly logistic regression models.

(A1) $$P(\sigma_{i}|{\vec{\sigma }}_{j\;\neq \;i};s)=\frac{\exp[h_{i}(s)+2\sum_{j\;\neq \;i}\sigma_{j}J_{ji}]\sigma_{i}}{1+\exp[h_{i}(s)+2\sum_{j\;\neq \;i}\sigma_{j}J_{ji}]}$$

where the functions *h*_*i*_(*s*) are generally expanded in basis functions of the stimulus as discussed in the main text. *N* logistic regression models are independently fit, one to each neuron's spikes. However, it should be emphasized that the model fit to neuron *i*'s spikes is conditioned upon the spikes of the other *N*\*i* neurons. Logistic regression models can be efficiently fit using iterative reweighed least squares techniques (Komarek and Moore, 2003; Komarek, 2004). The full computation times for pseudo-likelihood fitting were 9.5 s for the 20 neuron rat data, 40.5 s for the 42 neuron rat data, 19.1 s for the 20 neuron monkey data, 55.7 s for the 39 neuron monkey data and 34.2 s for the 20 neuron cat data.

Combining the parameters of the *N* logistic regression models gives the full set of *h*_*i*_(*s*) and the coupling matrix *J*. It should be noted that due to finite data sizes the fitted parameters will have some error, and the fitted coupling matrix *J* will not be exactly symmetric. It can be made symmetric by averaging it with its transpose: *J* ← 0.5(*J* + *J*^⊤^). A second point is that the above formalism will give a coupling matrix *J* with all-to-all connectivity. Although we did not pursue it in this paper, sparse coupling matrices can be obtained by applying either *p*-value or L1-regularization variable selection techniques as discussed in Gerhard et al. (2011) and Aurell and Ekeberg (2012), respectively. Still, for all our experimental data, the pseudo-likelihood fit Ising model had a higher test data log likelihood than an independent neuron model fit to the same training data and evaluated on the same test data indicating the presence of synchrony.

Figure A1 gives some examples of fitted parameters. The coupling matrix J (parameters in right column) have an intuitive meaning in terms of whether neurons exhibit correlated firing (positive values of J) or anti correlated (negative values). For example the cat data has mostly positive values for *J*_*ij*_ and hence these neurons exhibit a degree of “synchrony” while the rat spikes appear to be somewhat anti correlated (mostly negative couplings) and the monkey data has both positive and negative couplings. *J* also defines a weighted connectivity graph, which here exhibits all to all connectivity because we did not, in this paper, perform variable selection (see above).

In contrast, the fitted parameters quantifying the drive (left column) have little meaning in and of themselves, they attain their meaning by being multiplied by functions of the stimulus to produce the “external field” for each neuron, which is then passed through the logit function to obtain a firing probability for each neuron. That is, the fields are given by:

(A2) $$h_{n}(s)=\sum_{m\;=\;1}^{M}B_{m}(s)\beta_{nm}$$

and we have plotted histograms of the fitted parameters β in the left column. Note that it is not only the fields *h*_*n*_(*s*) that govern the firing rate, there is crosstalk with the coupling matrix *J*. Still this gives us some insight into why the drive parameters β are often negative and large, i.e., firing probabilities are small. The specific shapes of the drive parameter distributions can be understood in the context of the specific basis functions they are multiplied by.

For the rat data (upper left) we used a series of Zernike polynomials (Equation 20). The first term of this series is a constant equal to 1 and the parameters with large negative values are multiplied by these constants and are largely responsible for the low firing rates of the hippocampal neurons. The other parameters peaked around zero are both positive and negative. These multiply higher order Zernike polynomials and govern modulation of the neurons firing probabilities, about their means, as a function of the rat's position. In contrast, the monkey and cat data used B spline basis functions which depended on the time since trial onset, and in the case of the cat, grating direction. These splines are similar in shape to PSTH bins and therefore they are almost all negative so as to produce negative fields *h*_*n*_(*s*) and low firing probabilities. A few parameters are positive because the spline functions overlap. Fundamentally however, the meaningful quantity is the time varying firing probability, examples of which we gave in Figures 6, 8, and 10.

### Appendix B: partition function estimation via importance sampling

The idea behind importance sampling (Bishop, 2007; Salakhutdinov, 2008) is to sample from a distribution which is “close” to the Ising model and use those samples to calculate the *ratio* between the Ising partition function and that of the proposal (sample) distribution. Since correlations between neurons are weak (generally) we used an independent neuron model as the proposal distribution. That is, we independently fit to each neuron *i*'s spikes logistic regression models of the form.

(B1) $$P^{\text{indep}}(\sigma_{i}|s)=\frac{e^{f_{i}(s)\sigma_{i}}}{1+e^{f_{i}(s)}}$$

Here, the *f*_*i*_(*s*) have the same functional form as the *h*_*i*_(*s*) of the Ising model, but may have different fitted parameters. e.g., if, as in section 3, $h_{i}(s)=\sum_{m=1}^{M}B_{m}(s)\beta_{mi}$ where the *B*_*m*_(*s*) are basis functions of the stimulus, then $f_{i}(s)=\sum_{m=1}^{M}B_{m}(s)\alpha_{mi}$ where α_*mi*_ ≠ β_*mi*_ necessarily.

The independent neuron proposal distribution is the product of these fitted logistic regression models and can be written as:

(B2) $$P^{\text{indep}}(\sigma |s)=\frac{e^{\vec{f}(s)\cdot \vec{\sigma }}}{Z^{\text{indep}}(s)}$$

where

(B3) $$Z^{\text{indep}}(s)=\prod_{i\;=\;1}^{N}\left(1+e^{f_{i}(s)}\right)$$

This distribution is very fast to sample from, because each neuron's spikes can be sampled independently. Denoting this set of samples as Ω then the Ising partition function is given by

(B4) $$Z^{\text{ising}}(s)=Z^{\text{indep}}(s)\frac{1}{\left|\Omega \right|}\sum_{\vec{\sigma }\;\in \;\Omega }e^{(\vec{h}(s)\;-\;\vec{f}(s))\cdot \vec{\sigma }\;+\;{\vec{\sigma }}^{⊤}J\vec{\sigma }}$$

We note that importance sampling works best if the proposal distribution is “close” to the distribution being approximated. More refined techniques such as *annealed* importance sampling (Neal, 2001) have been developed to deal with this issue but in our case we found them to provide negligible improvement, perhaps because the independent neuron distribution is often reasonably close to the Ising distribution.

### Appendix C: partition function estimation via deterministic approximations

Many deterministic approximations (such as mean field theories and other variational methods) replace the Ising distribution with a product of “simpler” functions which are more easily evaluated. Such methods are often nearly instantaneous to apply, but provide only a lower bound upon the partition function which can, as we demonstrated in the results, be a poor approximation. Practically speaking, many of these approaches require the solution of a set of self consistent equations via an iterative procedure. The solution is then fed into an expression for the partition function. In this paper we compared with three variational approaches: (1) Naive mean field theory, (2) TAP corrected mean field theory and (3) the Bethe approximation fit via loopy belief propagation. We also compared with a “low firing rate” approximation presented by Roudi et al. (2009a) which is obtained via a perturbative expansion. Extensive treatments of mean field theories and the Bethe approximation can be found in Opper and Saad (2001). Also see Roudi et al. (2009a,b) for neuroscience geared discussions. Below we list only the main results necessary to calculate partition functions.

#### C.1 “Naive” mean field theory

The simplest form of mean field theory replaces the fluctuations in the spikes by their mean rate, or in the physics terminology, their “magnetizations” *m*_*i*_. Usually the mean field Equations are written in terms of “spins” *s*_*i*_ ∈ {−1, 1} rather than in terms of spikes σ_*i*_ ∈ {0, 1}, and the magnetizations lie within the range −1 ≤ *m*_*i*_ ≤ 1. Therefore, we first transform the parameters $\vec{h}(s)$ and *J* (fit via pseudo likelihood in the {0, 1} convention) to the {−1, 1} convention. The conversions are easily obtained by writing down the Ising “energy,” changing variables and collecting terms to obtain:

(C1) $$\begin{array}{l} h_{i}(s)\to h_{i}(s)=\frac{h_{i}(s)}{2}+\sum_{j\;\neq \;i}\frac{J_{ij}}{2}\\ \;J_{ij}\to J_{ij}=\frac{J_{ij}}{4} \end{array}$$

The self consistent Equations to be solved can then be written as

(C2) $$m_{i}(s)=\tanh\left(h_{i}(s)+\sum_{j\;\neq \;i}J_{ij}m_{j}(s)\right)$$

and the partition function (for the {−1, 1}) convention is written as

(C3) $$\begin{array}{l} \log{ℨ}_{\text{MF}}(s)=-\sum_{i}[\left(\frac{1+m_{i}(s)}{2}\right)\log\left(\frac{1+m_{i}(s)}{2}\right)\\ \;+\left(\frac{1-m_{i}(s)}{2}\right)\log\left(\frac{1-m_{i}(s)}{2}\right)]\\ \;+\sum_{i}h_{i}(s)m_{i}(s)+\sum_{ij}J_{ij}m_{i}(s)m_{j}(s) \end{array}$$

This can then be simply converted back to the {0, 1} convention via

(C4) $$\log Z_{\text{MF}}=\log{ℨ}_{\text{MF}}+\sum_{i}\frac{h_{i}(s)}{2}+\sum_{ij}\frac{J_{ij}}{4}$$

#### C.2 Tap correction

The Thouless Anderson and Palmer (TAP) correction to naive mean field theory (Thouless et al., 1977; Opper and Saad, 2001) essentially subtracts off the effect (mediated by other spins) of spin *i* upon itself. In the neuroscience context, this correction comes into play for neurons with high firing rates. The self consistent Equations have the form:

(C5) $$m_{i}(s)=\tanh\left(h_{i}(s)+\sum_{j\;\neq \;i}J_{ij}[m_{j}(s)-J_{ij}(1-m_{j}^{2}(s))m_{i}(s)]\right)$$

and the partition function becomes

(C6) $$\begin{array}{l} \log{ℨ}_{\text{TAP}}(s)=-\sum_{i}[\left(\frac{1+m_{i}(s)}{2}\right)\log\left(\frac{1+m_{i}(s)}{2}\right)\\ \;+\left(\frac{1-m_{i}(s)}{2}\right)\log\left(\frac{1-m_{i}(s)}{2}\right)]\\ \;+\sum_{i}h_{i}(s)m_{i}(s)+\sum_{ij}J_{ij}\\ \;\left[m_{i}(s)m_{j}(s)+\frac{1}{2}J_{ij}(1-m_{i}^{2}(s))(1-m_{j}^{2}(s))\right] \end{array}$$

which is then converted back to the {0, 1} convention in the same way as with Naive mean field theory. It should be noted that naive mean field theory and the TAP correction are merely the first two terms obtained when the Gibbs free energy is expanded in a Taylor series with respect to the inverse temperature [see chapter 3 of Opper and Saad (2001)].

#### C.3 Bethe approximation

The Bethe approximation is exact if the couplings have a “tree like” topology, that is, there are no loops in the coupling matrix *J*. In this case, the Ising joint probability density can be factored into a product of one and two node marginals (Opper and Saad, 2001; Ricci-Tersenghi, 2012). In cases where there are loops (such as ours) the Bethe approximation simply ignores this fact. Assuming that one can estimate the one and two node marginals, then the partition function can be written as:

(C7) $$\begin{array}{l} \log Z_{\text{Bethe}}(s)=\sum_{(ij)\;\in \;E}\sum_{\sigma_{i},\sigma_{j}}b_{ij}(\sigma_{i},\;\sigma_{j}|s)\\ \;[\log(b_{ij}(\sigma_{i},\sigma_{j}|s)-h_{i}(s)\sigma_{i}-h_{j}(s)\sigma_{j}-\sigma_{j}J_{ij}\sigma_{i}]\\ \;+\sum_{i}\left(1-q_{i}\right)\sum_{\sigma_{i}}b_{i}(\sigma_{i}|s)[\log b_{i}(\sigma_{i}|s)-h_{i}(s)\sigma_{i}] \end{array}$$

In the above the sum over (*ij*) ∈ *E* refers to a sum over graph *edges* and σ_*i*_ and σ_*j*_ are summed over their possible values of σ_*i*_ ∈ {0, 1} and *q*_*i*_ is the number of edges attached to node (neuron) *i*. The *b*_*i*_(σ_*i*_|*s*) and *b*_*ij*_(σ_*i*_, σ_*j*_|*s*) are the *estimated* one and two node marginal probabilities, usually referred to as “beliefs” in the literature. These are usually obtained using an iterative technique called *belief propagation* (Pearl, 1988; Yedidia et al., 2003, 2005; Watanabe and Fukumizu, 2009) which is a special case of the more general *expectation propagation* algorithm for marginal estimation (Minka, 2001). Chapter 3, sections 7–8 of Opper and Saad (2001) written by Jonathan Yedidia gives a particularly clear and concise discussion of the Bethe approximation and the belief propagation algorithm. Further details can be found in chapter 22 of Murphy (2012) which gives further algorithmic details, addresses issues of convergence when the graph has loops (loopy belief propagation) and also discusses the connection with expectation propagation. Although loopy belief propagation algorithms are known to converge unreliably (Pearl, 1988; Murphy, 2012), we were able in our case to get the algorithm to converge by using synchronous updates, a “damping factor” of 0.1 and by normalizing the messages to sum to 1 at each iteration. See section 22.2.4 of Murphy (2012) for discussion of these techniques.

#### C.4 “Low firing rate” approximation

In Roudi et al. (2009a), Roudi and colleagues derive a perturbative expansion of the partition function which applies in the low firing rate limit. After defining the quantity

(C8) $$Z_{0}(s)=\prod_{i=1}^{N}\left(1+e^{h_{i}(s)}\right)$$

they show that *Z* may be approximated by an expansion

(C9) $$\begin{array}{l} \frac{Z(s)}{Z_{0}(s)}-1\approx \sum_{i<j}\phi_{ij}\delta_{i}(s)\delta_{j}(s)\\ \;+\sum_{i<j<k}[\phi_{ij}\phi_{ik}+\phi_{ij}\phi_{jk}+\phi_{ik}\phi_{jk}\\ \;+\phi_{ij}\phi_{ik}\phi_{jk}]\delta_{i}(s)\delta_{j}(s)\delta_{k}(s) \end{array}$$

where δ_*i*_(*s*) is the stimulus dependent firing probability of neuron *i* and ϕ_*ij*_ = *e*^2*J*_*ij*_^ − 1. Note that (Roudi et al., 2009a) also includes 3rd order coupling terms which we have dropped here because we only consider pairwise interactions in this paper.
